# O.3.2-7 The health outcomes of a combined lifestyle intervention for adults with overweight

**DOI:** 10.1093/eurpub/ckad133.152

**Published:** 2023-09-11

**Authors:** Jitske Makaske, Kirsten Verkooijen, Kristina Thompson, Annemarie Wagemakers

**Affiliations:** Wageningen University and Research, The Netherlands; jitske.makaske@wur.nl; Wageningen University and Research, The Netherlands; jitske.makaske@wur.nl; Wageningen University and Research, The Netherlands; jitske.makaske@wur.nl; Wageningen University and Research, The Netherlands; jitske.makaske@wur.nl

## Abstract

**Purpose:**

Combined lifestyle interventions aim to reduce overweight and increase health in people at risk of developing overweight related diseases by helping participants to develop and maintain a healthy lifestyle. This study examined the health outcomes of a combined lifestyle intervention called X-Fittt GLI, which is imbedded in Dutch fitness centers. The intervention consists of 3 months intensive guidance regarding physical activity and nutrition, followed by 21 months of aftercare.

**Methods:**

Mixed methods were applied. Quantitative data were collected among 559 participants, including body measurements and quality of life assessed at the start, at one year, and at two years after the start. Paired samples t-tests were conducted to examine significant differences between the time points (Bonferroni correction applied). Additionally, semi-structured interviews were conducted with 12 participants about barriers and facilitators to maintain physical activity and healthy eating. Results were analyzed using thematic analysis.

**Results:**

After two years, participant’s weight (-3 kg), BMI (-1 kg/m2), and waist circumference (-4 cm) were significantly reduced compared to the start of the intervention. No effects were found for blood pressure, VO2-max, and quality of life. The largest weight effects were found after one year (-5.1 kg). Barriers influencing maintenance of physical activity were decreased physical or mental health, closure of the fitness center, and lack of time, while facilitators were group lessons, a nice atmosphere at the sports center, awareness, and enjoying exercising. Barriers to maintain a healthy diet were emotions and social activities, while facilitators were the food app and kitchen scale, seeing results, and a balanced diet.

**Conclusions:**

The combined lifestyle intervention X-Fittt GLI showed positive health outcomes. However, the effects are modest and decline during the second year of the intervention. More (social and/or mental) support during the aftercare period may increase the chances that participants maintain their newly adopted healthy routines.

